# Neuromelanin-targeted ^18^F-P3BZA PET/MR imaging of the substantia nigra in rhesus macaques

**DOI:** 10.1186/s13550-025-01297-5

**Published:** 2025-11-24

**Authors:** Hongyan Feng, Ning Tu, Ke Wang, Xiaowei Ma, Zhentao Zhang, Zhongchun Liu, Zhen Cheng, Lihong Bu

**Affiliations:** 1 Nuclear Medicine and PET-CT/MRI Center, Renmin Hospital of Wuhan University, 99Zhangzhidong Road, Wuchang District, Wuhan, Hubei 430060 China; 2 Department of Nuclear Medicine, The Second Xiangya Hospital, Central South University, Changsha, Hunan China; 3 Department of Neurology, Renmin Hospital of Wuhan University, Wuhan, Hubei China; 4 Department of Psychiatry, Renmin Hospital of Wuhan University, Wuhan, Hubei China; 5 State Key Laboratory of Drug Research, Molecular Imaging Center, Shanghai Institute of Materia Medica, Chinese Academy of Sciences, Shanghai, China

**Keywords:** ^18^F-P3BZA, Neuromelanin, Sequential PET/MR, Substantia nigra, SWI

## Abstract

**Background:**

Neuromelanin is mostly located in dopaminergic neurons in the substantia nigra (SN) pars compacta, and can be detected by magnetic resonance imaging (MRI). It is a promising imaging-base biomarker for neurological diseases. We previously developed a melanin-specific probe N-(2-(diethylamino)-ethyl)-^18^F-5-fluoropicolinamide (^18^F-P3BZA), which was initially developed for the imaging of melanoma. ^18^F-P3BZA exhibited high levels of binding to the melanin in vitro and in vivo with high retention and favorable pharmacokinetics. In this study we further investigated whether ^18^F-P3BZA could be used to quantitatively detect neuromelanin in the SN in healthy rhesus macaques.

**Results:**

^18^F-P3BZA exhibited desired hydrophobicity with estimated log K ow 5.08 and log D7.4 1.68. ^18^F-P3BZA readily crossed the blood-brain barrier with brain transport coefficients (Kin) of 40 ± 8 µL g-1s-1. ^18^F-P3BZA accumulated specifically in neuromelanotic PC12 cells, melanin-rich melanoma cells, and melanoma xenografts. ^18^F-P3BZA binding was significantly higher in B16F10 cells vs. amelanotic SKOV3 cells (6.17 ± 0.53%IA vs. 0.24 ± 0.05%IA) and in pigmented PC12 cells vs. non-pigmented PC12 cells(7.09 ± 0.15%IA and 0.29 ± 0.05%IA).In the biodistribution study, ^18^F-P3BZA had higher accumulation in B16F10 tumors (6.31 ± 0.99%IA/g) than in SKOV3 tumors (0.25 ± 0.09%IA/g). Meanwhile, ^18^F-P3BZA uptake in B16F10 tumors could be blocked by excess cold ^19^F-P3BZA (0.81 ± 0.02%IA/g, 88% inhibition, *p* < 0.05). PET/MRI ^18^F-P3BZA provided clear visualization of neuromelanin-rich SN at 30–60 min after injection in healthy macaques. The SN to cerebella ratios were 2.7 and 2.4 times higher at 30 and 60 min after injection. In vitro autoradiography studies ^18^F-P3BZA exhibited high levels of binding to the SN of healthy human and almost no binding to the SN of Parkinson’s disease patient.

**Conclusion:**

^18^F-P3BZA PET/MRI clearly images neuromelanin in the SN, and may assist in the early diagnosis of neurological diseases associated with abnormal neuromelanin expression.

## Introduction

Neuromelanin (NM) is a dark insoluble pigment present in some neurons in the brain. It is particularly concentrated in dopaminergic neurons of the substantia nigra (SN), which is an essential structure in the midbrain that plays critical roles in learning and normal life [1]. NM plays a pivotal role in the brain by way of reducing cellular oxidative stress, due to its antioxidant, toxic metal-binding, and radicalscavenging properties. The studies verified that NM-pigmented dopaminergic neurons in the SN tissue related to in several neurodegenerative and psychiatric disorders, such as Parkinson’s disease (PD), other neurodegenerative disorders with parkinsonism and schizophrenia [2, 3]. Thus, the change of NM-pigmented dopaminergic neurons in the SN is a potential indicator of neurodegenerative and psychiatric disorders [4, 5]. Unfortunately, those patients’ clinical symptoms typically lag behind until nigrostriatal dopaminergic neurons are mostly destroyed. Hence early, sensitive, and specific strategies for quantitative detection of dopaminergic neuron viability may benefit the diagnosis, monitoring, and prognostic prediction of neurodegenerative and psychiatric disorders. NM may be as a useful biomarker contribute to the assessment of neuron viability in the SN.

In the last decade the assessment of NM integrity changes via NM-sensitive structural magnetic resonance imaging (MRI) has been investigated in diagnosis and monitoring neurodegenerative and psychiatric disorders progression [6–8]. NM integrity can be assessed from its contrast and volume by NM-sensitive MRI. Measurements of the loss of dopaminergic neurons in SNpc may provide new insight. Dopaminergic neurons in the SNpc contain NM, a product of dopamine auto-oxidation, which exhibit paramagnetic T1-shortening properties in MRI [9, 10]. NM has a strong affinity for iron, which is observed as low-intensity areas on T2-weighted images and as signal loss on T2∗-weighted images. Thus, MRI for detection of NM loss in SNpc could reflect decreased dopaminergic neuronal density for disease diagnosis. Molecular changes always occur before structural changes that the PET tracer binds specifically to molecular marker with much more sensitive than MRI [11]. The development of NM-targeted molecular imaging techniques may facilitate early detection of nigrostriatal degeneration [12]. Hybrid PET/MRI instruments have been developed, and yield enhanced image quality and diagnostic accuracy. A tracer for accurate assessment of NM level in the SN in vivo is thus crucial to assist the diagnosis and monitoring of disease in the early stage.

^18^F-AV-1451, a tau radiotracer, was found to “off-target” bind to NM in the SN and could be a surrogate marker of PD [13–15]. However, to date there is no NM-targeting PET radiotracer available to image depigmentation of the SN. In a previous study we developed a melanin targeted probe, N-(2-(diethylamino)-ethyl)-^18^F-5-fluoropicolinamide (^18^F-P3BZA) for the imaging of melanoma [16, 17]. The radiation dose, biodistribution, and preliminary clinical application of ^18^F-P3BZA have been investigated. ^18^F-P3BZA binds to melanin with high affinity and specificity, and it is also safe for human imaging and can be used for human melanoma detection. Considering the important role of NM in PD, in the current study we investigated the capacity of ^18^F-P3BZA to target NM in vitro and in vivo. PET/MRI of healthy rhesus macaques (*n* = 4) was performed to evaluate whether ^18^F-P3BZAPET could serve as a new strategy for imaging NM in the SN.

## Materials and methods

### Preparation of ^18^F-P3BZA

Radiosynthesis and characterization of ^18^F-P3BZA was performed as previously described [16, 18].

^18^F-P3BZA was purified via an improved solid phase extraction-based method as previously reported [19]. Briefly, a crude product-captured C18 light cartridge was eluted with 20 mL 0.01 M NH_4_HCO_3_ and 2 mL ethanol/0.01 M NH_4_HCO_3_ (1/4, v/v), yielding high radiochemical purity within 40 min.

### Lipophilicity and *in situ* brain perfusion assay

The lipophilicity of ^18^F-P3BZA was experimentally measured via the conventional shake-flask method at room temperature as reported previously [20]. Briefly, 10 µL ^18^F-P3BZA (750 kBq) was dissolved in 3.0 mL of a 1:1 mixture (v/v) consisting of n-octanol and phosphate-buffered saline (PBS; pH 7.4). After shaking for 20 min at room temperature and centrifuging the mixture at 5000 rpm for 5 min, 1mL aliquots of both layers were taken and measured in a γ-counter (PerkinElmer Wallac Wizard 1480 Gamma Counter, WALLAC, Turku, Finland). For the second extraction another 1-mL aliquot of the organic layer was mixed with 5.0 mL of a 2:3 mixture (v/v) of n-octanol and PBS, and was treated using the same procedure. The distribution coefficient (D) was calculated as (activity [cpm/mL] in noctanol)/ (activity [cpm/mL] in PBS, pH 7.4), and expressed as the decade logarithm (log D7.4). Log Kow = logarithmic n-octanol/water partition coefficient, and log D7.4 = log Kow corrected for ionization at pH 7.4.

The blood-brain barrier (BBB) permeability of ^18^F-P3BZA was assessed using in situ brain perfusion assays as previously described [21]. Briefly, 4-week-old female Sprague Dawley rats (*n* = 3) were anesthetized, heparinized (10,000 U/kg), and maintained at a stable body temperature of 37 °C. After opening the thoracic cavity, the cardiac ventricles were severed to prevent blood from entering the systemic circulation. The cannulated left common carotid artery was connected to a perfusion circuit. The cerebral vessels were prewashed with saline for 10–45 s to remove erythrocytes and plasma components. Then, to perfuse the ipsilateral cerebral hemispheres, warmed (37 °C) and oxygenated perfusate containing ^18^F-P3BZA was infused into the left common carotid artery at a rate of 5 mL/min under a pressure of 80–120 mmHg. The perfusion time was 10 min followed by a 2-min washout with non-radioactive saline to clear the ^18^F-P3BZA trapped in brain blood vessels (Q vas). The total volume of perfusate for 10 min perfusion was calculated. After perfusion the rat was sacrificed, the brain was harvested, and the meninges, choroid plexus, and brain-surface blood vessels were removed. The ipsilateral hemisphere was divided and placed into pre-weighed vials, then weighed. The total concentration of solute of interest in the brain was then determined via scintillation counting. The same volume of perfusion fluid was filled into vials, then gamma-counting was performed to assess the amount of ^18^F-P3BZA. The BBB transfer coefficient (Kin) of ^18^F-P3BZA was determined using the following equation, where C_pf_ = the concentration of ^18^F-P3BZA in the perfusate (mass drug/volume fluid), Q_tot_ = the total amount of ^18^F-P3BZA in the brain (mass/weight brain), V_v_ = brain vascular volume (mL/g), and T = perfusion time:

Kin = (Q_tot_ – V_v_Q_p_f) / C_pf_T.

### *In vitro* cell uptake and NM content

To determine whether ^18^F-P3BZA specifically binds to NM, in vitro measurement of melanin content of neuromelanotic PC12 cells was performed in parallel with cell uptake assays in accordance with previously reported methods [18, 22]. PC12 rat pheochromocytoma, melanotic B16F10 melanoma, and amelanotic SKOV3 ovarian cell lines were originally obtained from the American Type Culture Collection (Manassas, VA, USA). PC12 cells were treated with 50 µmol/L L-3,4dihydroxyphenylalanine (L-DOPA, Alfa Aesar, Ward Hill, MA, USA) for 24 h to generate a model for NM synthesis [23]. B16F10 melanoma cells were used as melanin-positive controls, and amelanotic SKOV3 ovarian cells were used as melanin-negative controls. To test the binding capacity of ^18^F-P3BZA, 5 × 10^5^ cells were plated in 12-well plates and cultured overnight. The cells were then incubated with ^18^F-P3BZA (37 kBq per well) at 37 °C for 15, 30, 60, and 120 min, with or without blocking with excess cold ^19^F-P3BZA (10 µg per well) at the 60 min timepoint. Cellular uptake was expressed as the percentage of total applied radioactivity (% of applied activity, %IA). NM content was expressed as *A*_*405*_ per milligram of protein.

### *Ex vivo* biodistribution for ^18^F-P3BZA binding test in melanotic and amelanotic xenografts

All animal experiments were performed in accordance with a protocol approved by the Animal Care and Use Committee of Renmin Hospital, Wuhan University. Laboratory-bred female rhesus macaques aged 4.0–5.5 years were purchased from Hubei Tianqin Biotechnology Co. Ltd. (Wuhan, China). Female SD rats aged 6–8 weeks and female athymic nude mice (nu/nu) aged 4–6 weeks were obtained from HFK Bioscience Co., Ltd., (Beijing, China).

B16F10 or SKOV3 cells (1 × 10^7^) suspended in 100 mL of PBS were inoculated subcutaneously into the shoulder of nude mice. When tumors reached 0.5–0.8 cm in diameter the mice were used for ex vivo biodistribution studies. Mice were anesthetized via isoflurane inhalation (1–3% isoflurane in 1 L/min oxygen). Immediately after 1-h post-tracer injection with or without excess ^19^F-P3BZA (300 µg per mouse), mice bearing B16F10 tumors (*n* = 4/group, 2 groups) or SKOV3 tumors (*n* = 4/group, 1 group) were euthanized, and various organs were explanted and assayed for ^18^F-P3BZA activity as previously described [24].

### PET/CT imaging

A clinical PET/CT scanner (General Electric Company; GE^®^, Discovery 710) was used in this study. The CT part has 64 rows and 128 slices, and was used with standard parameters (CT: 120 Kv, 120–150 mA without contrast; 10 min/bed-PET-step of 15 cm). Reconstruction was performed with a 192 × 192 matrix and a voxel size of 2.73 × 2.73 × 3.27 mm^3^. The acquired PET data were reconstructed using Vue Point Fx (fully three-dimensional time-of-flight iterative reconstruction, VPFX). All images were exported in DICOM format for image feature extraction.

For dynamic scanning, rhesus macaques were anaesthetized with zolazepam hydrochloride (0.1 mL/kg) and 0.25 mg atropine sulfate, and 1/3 of zolazepam hydrochloride was administered after 40 min for maintenance during imaging. Monkeys were placed in the supine position and near the center of the field of view of the scanner. ^18^F-P3BZA (74-111MBq) was then injected via a tail vein, and scans were promptly acquired (12 × 10 s, 6 × 30 s, 4 × 60 s, 2 × 90 s, 4 × 2 min, 4 × 5 min, 2 × 10 min, total of 30 frames). Throughout baseline PET scans, blood samples (300 µL) were collected via the femoral vein at 5, 10, 20, 40, and 60 min and were analyzed for stability and biodistribution of ^18^F-P3BZA in plasma. For static scanning, monkeys were injected with ^18^F-P3BZA (74-111MBq) via a lower extremity vein and images were acquired after 15, 30, and 60 min.

### MRI imaging

MRI was performed immediately on a 3.0T MR (Discovery750W Silent-MR 3.0T, General Electric Company; GE^®^) after the last PET imaging. T1 BRAVO and T2-Flair sequences were acquired with TR/TE 2300/2.98 ms for T1 scanning and 9000/145 ms for T2-Flair scanning, section thickness 1.2 mm with no intersection gap, voxel size 256 mm, and a 256 × 256 matrix. NM-magnetic sensitive weighted imaging (NM-SWI) sequences were acquired with TR/TE 550/11, echo chain length 3, NEX 5, section thickness 1.2 mm with no intersection gap, voxel size 256 mm, and a 448 × 311 matrix. The NM-SWI sequence was located in the midbrain region parallel to the anterior-posterior associative plane. The MRI scans were then imported to the GE Workstation AW 4.6 and registered with PET images for further analysis.

### Autoradiography of human brain tissue samples

Healthy human and PD patient brain tissue containing SN samples were obtained from Stanford/NIA Brain Bank. Coronal Sect. (100 μm thick) containing SN were incubated for 50 min at room temperature with 4 MBq ^18^F-P3BZA in 200 mL of PBS (pH 7.4, 10 mM), washed with PBS three times for 5 min each, then exposed to ^18^F-sensitive storage phosphor screens (Perkin Elmer, USA) for 24 h at 4 °C. The image plates were analyzed using a Typhoon 9410 Variable Mode Imager (Amersham Biosciences, USA) [25, 26]. Brain slices adjacent to those used for autoradiography studies were stained with Fontana-Masson stains to verify the presence of NM [18].

### Statistics

Quantitative data from PET imaging were analyzed and plotted with GraphPad Prism 7.0 (GraphPad Software Inc., USA). Means were compared using Student’s *t* test. *p* < 0.05 was considered statistically significant.

## Results

### Capacity of ^18^F-P3BZA to cross the BBB

Lipophilicity is considered an important physicochemical property, and is often associated with a capacity to cross the BBB. ^18^F-P3BZA exhibited high hydrophobicity, log Kow 5.08, and log D7.4 1.68. In the in situ brain perfusion experiments in rat ^18^F-P3BZA readily crossed the BBB with brain transport coefficients (Kin) of 40 ± 8 µL g-1s-1. High-performance liquid chromatography analysis confirmed the molecular integrity of the ^18^F-P3BZA dissolved in perfusion fluid after in situ cerebral perfusion.

### Correlation between ^18^F-P3BZA uptake and melanin content

**Fig. 1:**
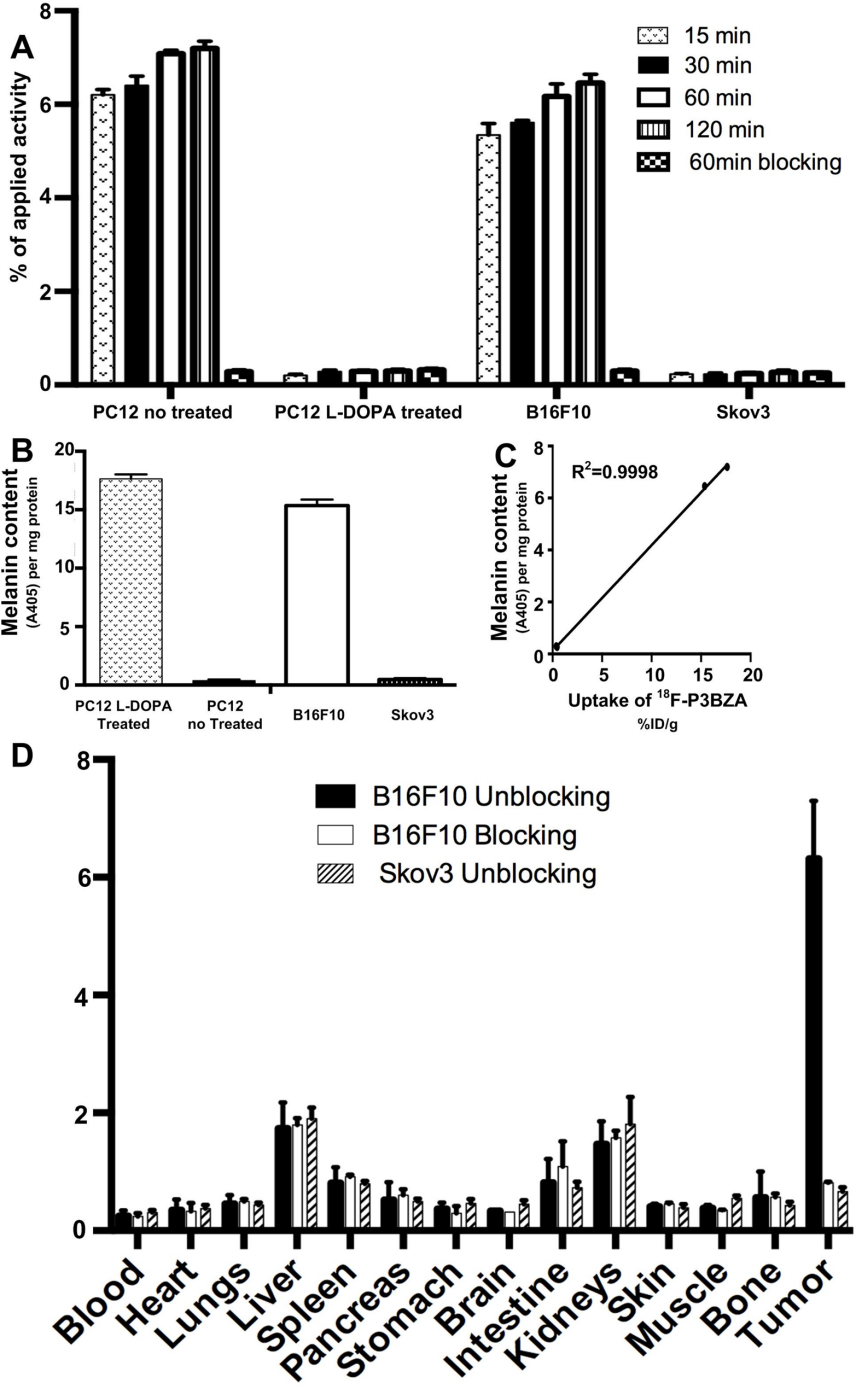
In vitro, in vivo, and ex vivo ^18^F-P3BZA tests. (**A**) Time-dependent uptake of ^18^F-P3BZA after incubation for 15, 30, 60, and 120 min in PC cells with or without pretreatment with L-DOPA for 24 h. B16F10 cells and SKOV3 cells were used as controls. Results shown are means of triplicate measurements ± standard deviations. (**B**) Quantitative analysis of melanin levels in different cell types (*n* = 4). (**C**) Correlational analysis of ^18^F-P3BZA uptake and melanin content in B16F10 neuromelanotic cells. Data are represented as means ± standard deviations. (**D**) Biodistribution of ^18^F-P3BZA in mice bearing subcutaneously xenotransplanted B16F10 or SKOV3 cells with or without blocking with excess cold ^19^F-P3BZA

*In vitro* cellular uptake experiments ^18^F-P3BZA accumulated in neuromelanotic PC12 cells pretreated with L-DOPA effectively (7.09 ± 0.15%IA) at 60 min post-incubation but not in aneuromelanotic PC12 cells (0.29 ± 0.05%IA) (*p* < 0.05). Similarly, ^18^F-P3BZA exhibited a much higher level of accumulation in positive control melanotic B16F10 cells (6.17 ± 0.53%IA) than in negative control amelanotic SKOV3 cells (0.24 ± 0.05%IA) at 60 min. After blocking with excess ^19^F-P3BZA, the uptake of ^18^F-P3BZA dropped to 0.28 ± 0.07%IA in neuromelanotic PC12 cells and 0.29 ± 0.08%IA in melanotic B16F10 cells (Fig. 1A). There were no differences in ^18^F-P3BZA uptake in aneuromelanotic PC12 cells or amelanotic SKOV3 cells with or without blocking.

In melanin content assays the amounts of NM in neuromelanotic PC12 cells were much higher than those in control aneuromelanotic PC12 cells (17.64 ± 0.77 *A*_*405*_/mg protein vs. 0.38 ± 0.18 *A*_*405*_/mg protein) (Fig. 1B). ^18^F-P3BZA uptake at the 60-min timepoint in B16F10 neuromelanotic cells was linearly correlated with their relative NM content levels (Fig. 1C, *R*^2^ = 0.9998).

### Targeting efficiency and biodistribution of ^18^F-P3BZA in mice

^18^F-P3BZA had clearly higher accumulation in B16F10 tumors (6.31 ± 0.99%IA/g) than in SKOV3 tumors (0.25 ± 0.09%IA/g) and in blood (0.25 ± 0.09%IA/g). When excess cold ^19^F-P3BZA was coinjected, ^18^F-P3BZA uptake in B16F10 tumors at 1 h post-injection was significantly reduced (0.81 ± 0.02%IA/g, 88% inhibition, *p* < 0.05). Renal and hepatic levels of ^18^F-P3BZA uptake were significant, suggesting both renal-urinary and hepatic-biliary routes of tracer clearance (Fig. 1D).

### PET and MRI of rhesus macaques

**Fig. 2:**
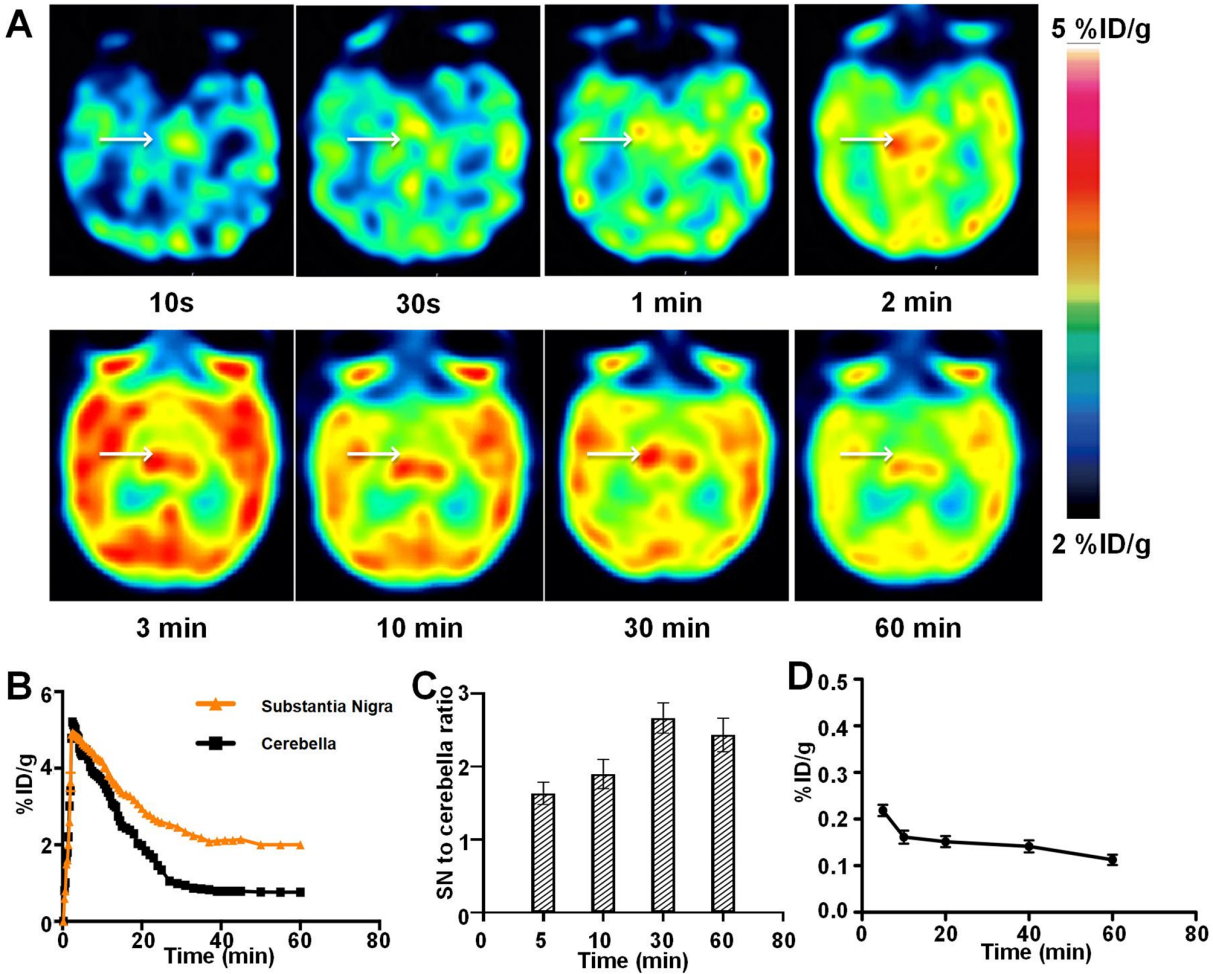
Dynamic PET of rhesus macaque brains and quantitative image analysis. (**A**) Axial decay-corrected dynamic PET images obtained after injection of ^18^F-P3BZA into healthy rhesus macaques. Arrows indicate the SN. (**B**) Time-activity curve derived from PET studies. (**C**) SN to whole brain uptake ratios at different imaging times. (D) Time-activity curve derived from blood samples.

**Fig. 3:**
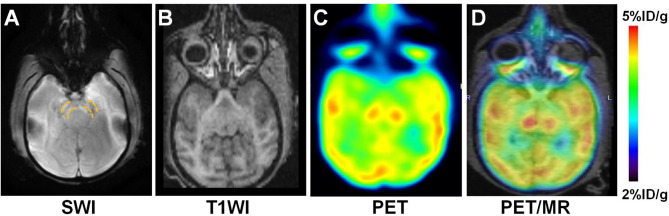
Static sequential PET/MRI of the SN region in rhesus macaques. (**A**) SWI MRI. (2) T1WI MRI. (**C**, **D**) Representative decay-corrected axial ^18^F-P3BZA PET and PET/MRI (white arrows indicate the SN)

Dynamic PET images provided visual evidence that ^18^F-P3BZA could rapidly cross the BBB and accumulate in the SN. On dynamic PET imaging the SN was visible approximately 1 min after injection of the tracer, and was clearly visualized with high contrast 30–60 min after injection. Similarly, the time-activity curve of the SN from dynamic PET scans indicated that ^18^F-P3BZA uptake in the SN peaked at 4.84 ± 0.22%ID/g within the first 3 min by first pass, then gradually decreased to 3.02 ± 0.14%ID/g at 30 min after injection, reached a plateau at 30 min, and remained stable for up to 60 min, providing an optimum time window for static PET imaging (Fig. 2A and B). The respective SN to cerebella ratios 5, 10, 30, and 60 min after injection were 1.6, 1.9, 2.7, and 2.4 times higher (Fig. 2C). The best imaging time for SN ^18^F-P3BZA PET was 30–60 min after injection. There were low levels of ^18^F-P3BZA in the blood (Fig. 2D). On static PET/MRI scans, when focusing on the SN area, low signal was observed on SWI images overlaid with the high PET signal area, indicating a high concentration of NM in the SN (Fig. 3).

### Autoradiography and fontana-masson staining of postmortem human brains

**Fig. 4:**
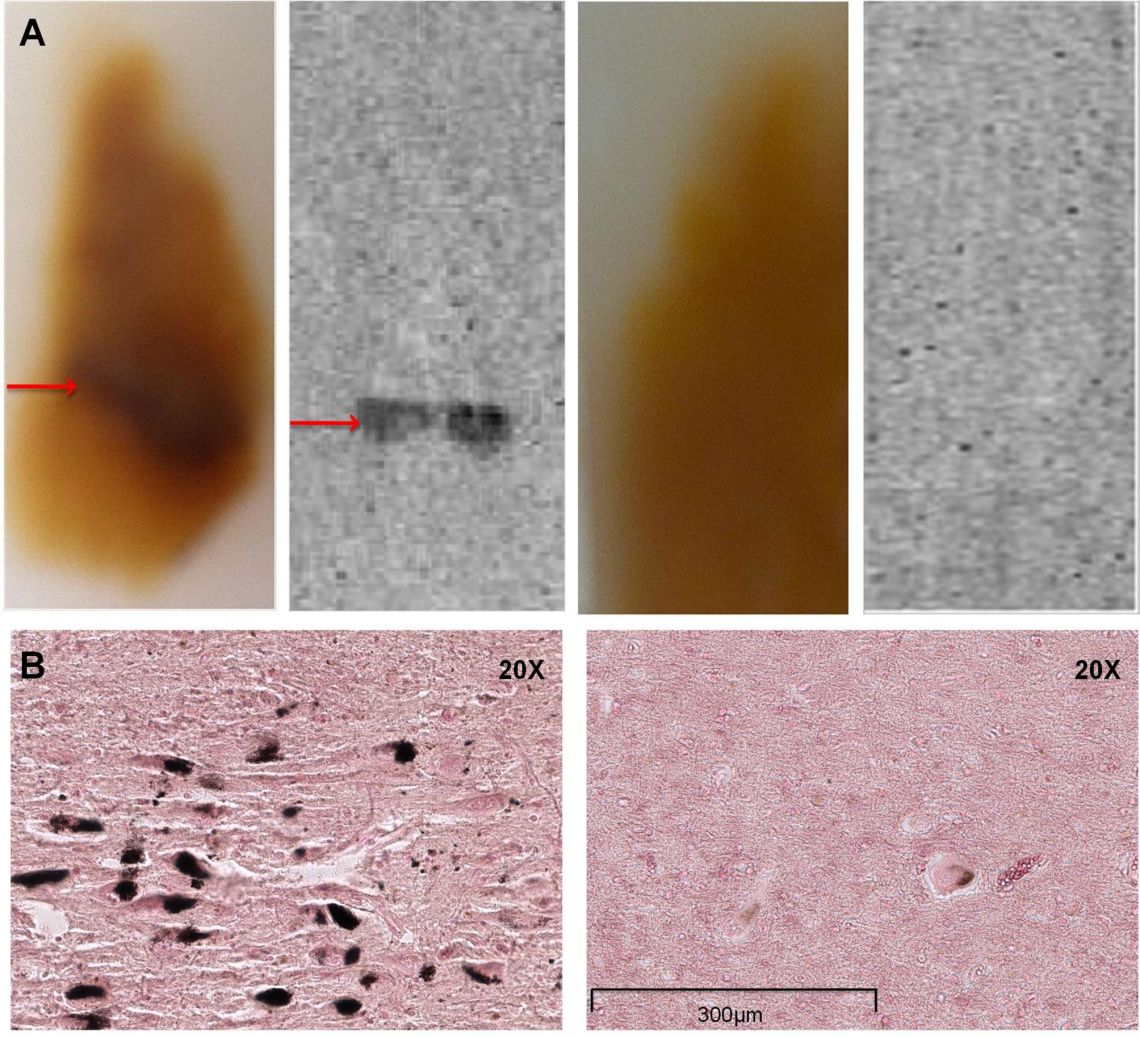
Ex vivo autoradiography, healthy human and PD patient brain tissue were obtained from the Stanford/NIA Brain Bank. (**A**) Axial autoradiography of human SN: healthy (left) vs PD(right). Red arrow indicates SN. (**B**) Representative photomicrographs of Fontana-Masson-stained SN sections: healthy(left) vs PD (right).

Ex vivo autoradiography clearly indicated that ^18^F-P3BZA mainly accumulated in the SN of healthy human, and much less accumulated in the SN of PD patient (Fig. 4A). Pathology staining revealed the NM content in the SN of healthy human and was consistent with specific uptake of ^18^F-P3BZA in the SN (Fig. 4B).

## Discussion

In the past few years ^18^F-P3BZA has been developed as a melanin-specific PET probe for melanoma diagnosis and melanotic cell trafficking [16–18]. A radiochemistry procedure has been optimized for automatic synthesis of ^18^F-P3BZA, and radiation dosimetry in healthy volunteers has been estimated. In a preliminary clinical study ^18^F-P3BZA exhibited high binding affinity with the melanin in melanoma, and was useful for melanoma diagnosis and management [16]. Given the strong capacity of ^18^F-P3BZA to facilitate quantitative imaging of melanin in vivo, in the current study we evaluated the capacity of ^18^F-P3BZA to enable imaging of NM in the SN in monkey brain. Such a study may lay a foundation for imaging NM in humans, and thus estimating the function of SN in patients with diseases associated with NM abnormality. ^18^F-P3BZA was structured and purified via an improved solid phase extraction-based method [19]. This purification method is much faster than previously described methods and it is low cost, thus potentiating the wider use of ^18^F-P3BZA in the future.^18^F-P3BZA specifically bound to NM in vitro, and it could pass through the BBB and accumulate in the SN efficiently.

*In vitro* experiments there was significantly increased ^18^F-P3BZA accumulation in PC12 cells after treatment with L-DOPA to produce NM, compared to non-treated PC12 cells. Binding of ^18^F-P3BZA to NM could be blocked by excess cold ^19^F-P3BZA, indicating that ^18^F-P3BZA bound specifically to NM. In cellular uptake assays using melanin-rich B16F10 cells and amelanotic SKOV3 cells there was a positive correlation between ^18^F-P3BZA uptake and melanin content. In in vivo biodistribution experiments in mice high levels of ^18^F-P3BZA accumulated in melanotic B16F10 tumors, and the uptake could be blocked by excess ^19^F-P3BZA, whereas there was very low uptake in amelanotic SKOV3 tumors. Together these results indicate specific melanin targeting of ^18^F-P3BZA, which is a valuable foundation for further in vivo imaging of NM in the SN. In ex vivo autoradiography experiments ^18^F-P3BZA specifically accumulated in NM in the SN of normal human brain tissue slides, highlighting the potential of ^18^F-P3BZA for evaluating NM in the SN.

*In vivo* studies sequential monkey brain PET/MRI was successfully performed. ^18^F-P3BZA accumulated in the SN for a long period of time (up to 60 min) and delineated the SN clearly, even though other brain areas also exhibited varying levels of probe uptake. The NM concentration in the SN can be analyzed by drawing a region of interest over the SN based on the edge depicted on MRI. NM is also a major absorber of iron, and inhibits the generation of superoxide free radicals against iron-mediated neurotoxicity. Thus, T1-weighted MRI and NM-SWI can reflect changes in NM via natural iron contrast [14]. Therefore, the combination of ^18^F-P3BZA PET and MRI is a novel imaging strategy to estimate NM pigmentation in the SN.

Imaging of nigrostriatal dopamine pathway degeneration in neurodegenerative disorders plays a major role in clinical practice and in clinical research. Many PET probes that target presynaptic dopamine transporters, the striatal aromatic amino acid decarboxylase, and the vesicular monoamine transporter have been used to the diagnosis and differential diagnosis neurodegenerative and to rationalize treatment with dopamine replacement agents [27]. However, several concerns remain. First, to preserve decreased striatal dopamine, additional dopamine dysfunction in remaining terminals occurs as an adaptive mechanism. Consequently, dopaminergic dysfunction imaging techniques tend to overestimate the true degree of striatal dopamine loss. Second, dopaminergic dysfunction imaging cannot reliably discriminate different type of neurodegenerative that can affect striatal dopamine function, such as PD, multiple system atrophy, progressive supranuclear palsy, and corticobasal degeneration. Third, dopaminergic dysfunction occurs when neurodegenerative patients have lost most of the nigrostriatal dopaminergic neurons, so early diagnosis of neurodegenerative is difficult. Measurements of the loss of dopaminergic neurons in the SN may provide new insight into overcoming these difficulties. NM in the SN is markedly decreased after the loss of dopaminergic neurons during neurodegenerative progression. Thus, NM in the SN may be an alternative target for early neurodegenerative imaging. Lastly, we successfully used ^18^F-P3BZA PET/MRI to evaluate NM features in the SN in monkeys. A clinic trial is required in the future to acquire data from either healthy people or neurodegenerative patients, to investigate concordance between the loss of nigral ^18^F-P3BZA accumulation on PET and nigrostriatal dopaminergic degeneration.

## Conclusion

NM in the SN can be visualized and quantified by ^18^F-P3BZA PET/MRI, which as a new strategy for imaging NM may be beneficial for neurological diseases associated with abnormal neuromelanin expression. 

## Data Availability

The datasets used during the current study are available from the corresponding author on reasonable request.
